# USING RISE IN CASES DIVERTING DEFENDANTS IN ELDER ABUSE SCENARIOS OUT OF THE CRIMINAL SYSTEM AND INTO TREATMENT

**DOI:** 10.1093/geroni/igad104.1157

**Published:** 2023-12-21

**Authors:** M T Connolly, Page Ulrey, Patricia Kimball, Geoff Rogers, Stuart Lewis, David Burnes

**Affiliations:** University of Southern California, Los Angeles, California, United States; King County Prosecuting Attorney’s Office, Seattle, Washington, United States; Elder Abuse Institute of Maine, Brunswick, Maine, United States; City University of New York, New York City, New York, United States; Dartmouth College, Lebanon, New Hampshire, United States; University of Toronto, Toronto, Ontario, Canada

## Abstract

Criminal justice system interventions are a common response to elder abuse, neglect and exploitation (EA), but there is severely limited understanding of their efficacy in reducing EA or improving the well-being of older adults. A goal attainment scaling feasibility study revealed that APS clients’ goals often included getting help (like substance use or mental health treatment) for someone else, often family members harming them. Some APS and RISE clients reported reluctance to seek help or report harm, fearing that involvement of the criminal justice system might result in them losing control over their lives, losing a caregiver, being forced into a facility, or legal punishment of someone they care about. Given these findings, the RISE team engaged in a lengthy outreach and planning phase with state and local prosecutors, elder victim service providers, restorative justice experts, and researchers, to design a novel intervention, using RISE in conjunction with drug court, to divert cases involving substance using defendants alleged to have mistreated an older person. This session will describe the extensive planning and design process and provide a case study analysis, which suggests that a combination of drug court’s in-patient treatment for the defendant, services for the victim, and RISE’s focus on restorative justice approaches that repair breached relationships reduced harm and benefited the older person, the defendant, and their families. Implementation of RISE in partnership with the criminal justice system carries important implications for the integration of restorative justice practices in some EA cases.

